# Comparison of IOP Measurement by Goldmann Applanation Tonometer, ICare Rebound Tonometer, and Tono-Pen in Keratoconus Patients after MyoRing Implantation

**DOI:** 10.1155/2019/1964107

**Published:** 2019-05-09

**Authors:** Mahmoud Rateb, Mahmoud Abdel-Radi, Zeiad Eldaly, Mohamed Nagy Elmohamady, Asaad Noor El Din

**Affiliations:** ^1^Department of Ophthalmology, Faculty of Medicine, Assiut University, Assiut, Egypt; ^2^Tiba Eye Center, Assiut, Egypt; ^3^Department of Ophthalmology, Benha University, Benha, Egypt; ^4^Masa Eye Center, Benha, Egypt; ^5^Department of Ophthalmology, Al Azhar University, Cairo, Egypt

## Abstract

**Purpose:**

To evaluate the different IOP readings by Goldmann applanation tonometer (GAT), ICare rebound tonometer, and Tono-Pen in keratoconus patients after MyoRing implantation. To assess the influence of central corneal thickness (CCT) and thinnest corneal location (TCL) on IOP measurements by different tonometers.

**Setting:**

Prospective observational study was conducted in two private centers in Egypt from February 2015 to November 2016.

**Methods:**

Seventeen eyes of 10 patients suffering from keratoconus and who underwent MyoRing implantation were recruited. All subjects underwent GAT, ICare, and Tono-Pen IOP measurements in random order. Central corneal thickness and thinnest corneal location were assessed by Pentacam. Difference in mean in IOP readings was assessed by *T*-test. Correlation between each pair of devices was evaluated by Pearson correlation coefficient. The Bland–Altman analysis was used to assess intertonometer agreement.

**Results:**

Seventeen eyes (10 patients) were evaluated. The mean IOP reading was 13.9 ± 3.68, 12.41 ± 2.87, and 14.29 ± 1.31 mmHg in GAT, ICare, and Tono-Pen group, respectively. There was a significant difference between IOP readings by GAT/ICare and Tono-Pen/ICare (*p* value: 0.032 and 0.002, respectively) with no significant difference between GAT/Tono-Pen (*p* value: 0.554). Mean difference in IOP measurements between GAT/ICare was 1.49 ± 2.61 mmHg, Tono-Pen/ICare was 1.89 ± 2.15 mmHg, and GAT/Tono-Pen was −0.39 ± 2.59 mmHg. There was no significant correlation between the difference in IOP readings among any pair of devices and CCC or TCL. The Bland–Altman analysis showed a reasonable agreement between any pair of tonometers.

## 1. Introduction

Keratoconus (KC) is a progressive ectatic corneal disease characterized by corneal thinning associated with ametropia, mostly irregular astigmatism and myopia [1]. It usually occurs in the teenagers and affects both males and females [2]. The rigid gas permeable (RGP) contact lenses have been used to regularize the corneal surface in those patients, but the implantation of intracorneal ring segments (ICRS) is now gaining more popularity to achieve that purpose with resultant improved patient's visual acuity and quality [3].

Many types of ICRS are available, e.g., Intacts (Addition Technology Inc.), Ferrara Ring (Ferrara Ophthalmics Ltd.), and Keraring (Medicophacos Ltd.). However, Daxer et al. described the use of a full corneal intrastromal ring-MyoRing (Dioptex GmbH, Austria) to regularize the cornea especially in those patients with myopia and myopic astigmatism [4]. The MyoRing is made of polymethyl methacrylate (PMMA). It can be implanted either manually or more precisely with the use of corneal pocket software in Femto-laser devices. The ring is implanted into the corneal pocket with a diameter of nearly 8 mm, at a depth of 78% of the thinnest corneal pachymetry [5, 6].

IOP measurement in patients with corneal diseases (KC and dystrophies) or after corneal refractive surgeries varies significantly and represents a real challenge for many ophthalmologists [7, 8]. IOP measurement is markedly affected by the central corneal thickness (CCT) and corneal curvature (CC) which show marked variation in patients with keratoconus and after implantation of corneal rings. Though Goldmann applanation tonometry (GAT) is the gold standard for IOP measurement by many ophthalmologists, its accuracy is questionable in such patients [9–11]. In order to reduce the effect of corneal parameters on IOP measurements, new tonometers with less corneal contact in eyes with keratoconus and/or implanted corneal rings have been developed [12–14].

In this study, we compared IOP readings recorded by three types of tonometers (Goldmann tonometer, Tono-Pen, and Impact rebound tonometer) in keratoconus patients who underwent MyoRing implantation.

### 1.1. Goldmann Tonometer

The Goldmann tonometer is based on the applanation principle where a force is used to applanate an area of 3.06 mm of the cornea. At this point, the corneal rigidity and tear film surface tension are equal and cancel each other; thus, the force of the tonometer in grams multiplied by 10 equals the intraocular pressure (IOP) of the eye in mmHg [15, 16].

### 1.2. Tono-Pen™ Tonometer

Tono-Pen tonometer is also based on the applanation principle, and the instrument consists of a plunger and an electronic transducer measuring the movements of the plunger that is opposed by the IOP. Four readings are recorded, and the mean is calculated for more accurate repeatable IOP readings. The Tono-Pen tonometer is thought to be more accurate in irregular corneas as it used a smaller area of the cornea compared to GAT and depends on an electronic endpoint; however, its readings are also affected with the corneal thickness [17, 18].

### 1.3. Impact Rebound Tonometer

The impact rebound tonometer is based on the indentation principle; sterile probe is fired forward into the cornea; the time taken for the probe to return to its resting position and the characteristics of the rebound motion with creation of induction current are used to calculate the IOP. The device has number of advantages such as being easy to use, portable, and can be used without anesthesia.

But on the other hand, based on its mechanism of action, its measurements are not that accurate especially in corneal scarring, and it is also influenced by the corneal thickness [19, 20].

## 2. Patients and Methods

This study is designed as a prospective cross-sectional study conducted at two private centers in Egypt in the period from February 2015 to November 2016. The study followed the tenets of the Declaration of Helsinki. Oral and written consents were obtained from each participant in this study.

Seventeen eyes of 10 patients (3 patients with unilateral MyoRing and 7 patients with bilateral rings) were included in the study. The inclusion criteria were keratoconic eyes with central or paracentral cones shown on Pentacam who underwent MyoRing insertion at least 6 months before examination with mean k-readings between 48 and 56 diopters. The exclusion criteria include previous corneal surgery or corneal collagen cross linking and other ocular pathologies or surgeries.

All patients underwent complete ophthalmic workup including medical history, UCVA, BCVA, slit lamp biomicroscopy, fundus examination, Pentacam, and IOP measurements with three types of tonometers (impact rebound tonometer, Tono-Pen, and Goldmann applanation tonometer).

All IOP readings were taken by the same doctor starting with the impact rebound tonometer, then Tono-Pen, and finally the Goldmann applanation tonometer with a 5-minute recovery time between each measurement.

Regarding the impact rebound tonometer, the patient was asked to look straight ahead at specific point and the doctor brought the tonometer with the tip of the probe around 6 mm from the central cornea, six IOP readings were recorded, and the average was automatically calculated.

After calibrating Tono-Pen, a drop of ocular anesthetic (benoxinate hydrochloride 0.4%) was instilled in the patient's lower fornix, and the device was applied perpendicular to the corneal surface and touched it at least 4 times until 4 valid readings were obtained with the average calculated.

Finally, the patient was seated comfortably at the slit lamp, another drop of local anesthetic was instilled, a fluorescein strip was inserted in the lower fornix shortly then removed and washed gently, blue filter was activated, and the sterile Goldmann prism head moved gently until it touched the cent of the cornea. The calibrated dial on the tonometer turned clockwise until the inner edges of the two fluorescein semicircle images just touch. Three consecutive GAT readings were recorded, and the average was calculated. At least 1-minute break between readings was used to diminish the tonographic effect of applanation tonometry.

## 3. Results

### 3.1. Demographics

Seventeen eyes (10 patients) were evaluated. There were 4 males (40%) and 6 females (60%). The mean age was 28.47 ± 3.84 years (23–36 years). All patients were diagnosed with keratoconus and underwent intracorneal MyoRing segment implantation. Mean central corneal thickness (CCT) was 484.65 ± 29.74 *μ*m (95% CI: 469.36–499.94 *μ*m). Mean thickness at thinnest corneal location was 466.35 ± 36.6 *μ*m (95% CI: 447.53–485.17 *μ*m).

### 3.2. IOP Measurements

The mean IOP reading obtained by GAT was 13.9 ± 3.68 mmHg (range: 8–20 mmHg; 95% CI: 12.01–15.79 mmHg), by Tono-Pen was 14.29 ± 1.31 mmHg (range: 12–17 mmHg; 95% CI: 13.61–14.96 mmHg), and by I-Care was 12.41 ± 2.87 mmHg (range: 9–17 mmHg; 95% CI: 10.93–13.88 mmHg).

Mean difference between GAT and Tono-Pen reading was 0.39 ± 2.59 mmHg (range: 4.85–3.35 mmHg; 95% CI: 1.73–0.94 mmHg) with no statistically significant difference between both readings (*p* value: 0.540). Mean difference in IOP measurements by GAT and ICare reading was 1.49 ± 2.61 mmHg (range: 2.30–6.35 mmHg; 95% CI: 0.15–2.83 mmHg) with statistically significant difference (*p* value: 0.032). Mean difference between Tono-Pen and ICare reading was 1.88 ± 2.14 mmHg (range: 2.0–4.5 mmHg; 95% CI: 0.78–2.99 mmHg) with also statistically significant difference (*p* value: 0.002).

### 3.3. Correlation Analysis

**Figure 1:**
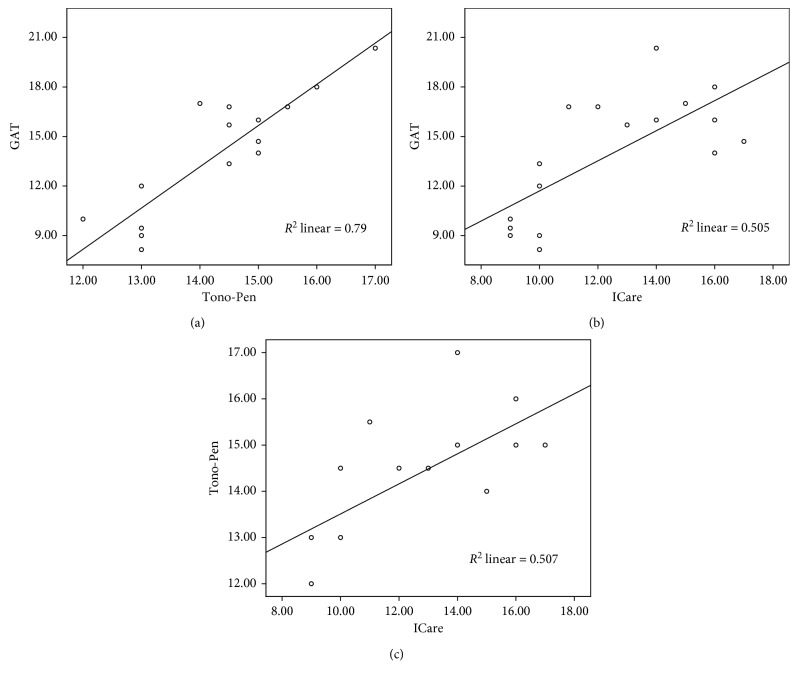
Linear regression analysis and scatter blot distribution of IOP measurements by different tonometers.

There was a strong positive correlation between all IOP measurement methods (Table 1). Linear regression analysis and scatter blot are shown in Figure 1. There was a strong association between all IOP readings by different tonometers.

**Figure 2:**
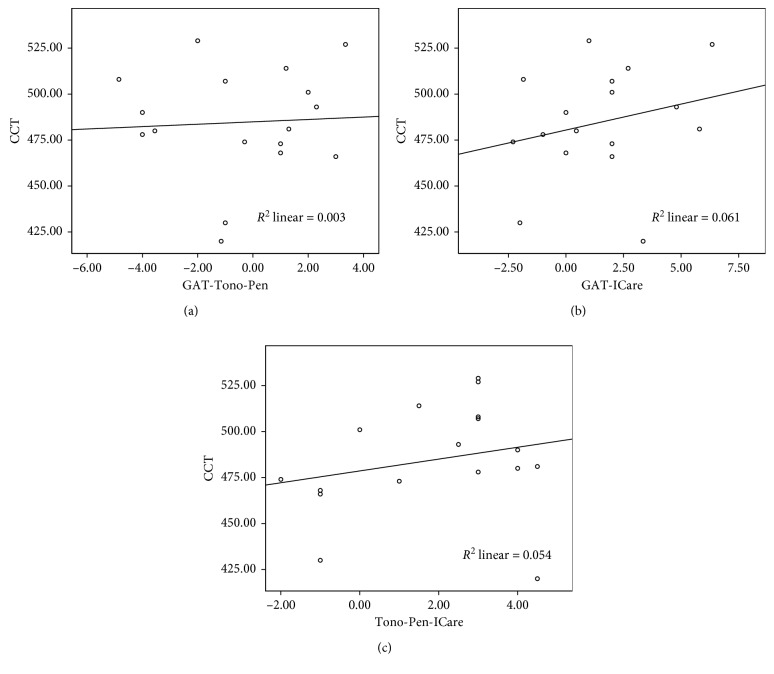
Linear regression analysis and scatter blot distribution of CCT and IOP measurements by different tonometers.

There was an insignificant correlation between CCT and the mean difference in IOP measurement by different methods (Table 2). Linear regression analysis and scatter blot are shown in Figure 2. There was a weak association between all IOP readings by different tonometers.

### 3.4. Bland–Altman Agreement Analysis

**Figure 3:**
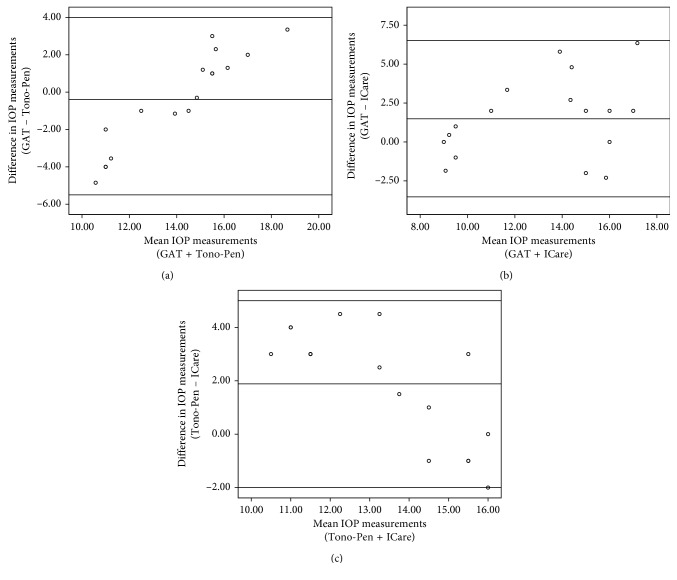
Bland–Altman plots of the agreement between different IOP measurements: (a) GAT vs. Tono-Pen, (b) GAT vs. ICare, and (c) Tono-Pen vs. ICare.

The Bland–Altman plots of the agreement between different IOP measurements are shown in Figure 3. The plots show the distribution of the difference in IOP measurement by two methods on the *y*-axis while the mean IOP value of both tonometers is represented on the *x*-axis.

The Bland–Altman scatter plot comparing all methods of IOP measurement showed a reasonable agreement between each two methods. The differences between corresponding measures, the standard deviation, and the 95% confidence interval are presented in Figure 3.

## 4. Discussion

Accurate IOP measuring after corneal surgeries is both challenging and vital: challenging due to changes in corneal thickness, curvature, and biomechanics and vital due to the risk of high IOP-related ocular complications when topical corticosteroids are used.

GAT was first introduced in 1957 and it is, till now, the most commonly used method for IOP because of its preciseness and easy use with low intraobserver and interobserver variability [21]. However, it may be influenced by corneal thickness that deviates from an idealized normal value and in the cases with corneas that are steeper, flatter, or more astigmatic than average as in KC [10, 11]. GAT requires the use of anesthetic eye drops and a slit lamp. The electronic applanation tonometers available, such as the Tono-Pen, also require the administration of a local anesthetic, but does not depend on a slit lamp [22]. The ICare tonometer is based on the induction-based rebound method with some merits in the form of portability, ease of use with good reproducibility, no need for topical anesthesia or slit lamp [23].

MyoRing is made of PMMA and, therefore, reinforces the cornea, resulting in alteration in the shape and the biomechanics of the cornea. MyoRing does not remarkably alter CCT [24] which is known to significantly impact GAT IOP measurements [25]. In this study, we compared three different methods of IOP measurement after MyoRing implantation in cases of KC. About 6 months are needed for ICRS to exert its maximal effect on the cornea because of the corneal viscoelasticity, [26] and so, we included cases with MyoRings implanted at least 6 months before examination.

We found that the mean IOP obtained by ICare was significantly lower than those by GAT and Tono-Pen; this is in accordance with results of previous studies [27–29]. The difference was approximately 2 mmHg, and so, the statistically significant differences found between GAT and ICare were not considered clinically relevant [27]. We consider the change in corneal rigidity after MyoRing implantation is a possible reason; however, the ICare tonometer was found to be lower. There was a strong positive correlation between IOP measurements by the three methods. This was the same in some previous studies [28–30].

One of 17 eyes differences between GAT and ICare seem to be 2 SD of mean error (Figure 3). One of 17 eyes differences between Tono-Pen and ICare seem to be 2 SD of mean error (Figure 3). Hence, 5.88% of eyes presented higher differences between GAT and ICare, but no eye showed difference outside boundary limits (95% confidence interval). This percentage is less than that found in other studies, in which agreement between GAT and other tonometers was investigated. Ceruti et al. found that the mean difference in postkeratoplasty patients was positive, and in 6.5% of the patients, the values fall outside boundary limits (95% confidence interval) [31].

In our study, there was no significant correlation between CCT and the mean difference in IOP measurement by different methods. This was the case in the study by Klamann et al., in which they evaluated the effect of CCT of keratoconic corneas on IOP measurements as measured by four different techniques, and they found that ICare and GAT were found to be independent of CCT in keratoconic corneas [27]. This similarity between our results and theirs may be because both studies investigated keratoconus cases.

On the other hand, Brusini et al. compared the IOP readings with ICare tonometer and GAT. They found that CCT change of 10 mm resulted in a lower ICare reading which is of 0.7 mmHg [29]. This difference between our results and their finding may be because they investigated cases with primary open-angle glaucoma (POAG).

In conclusion, there is a strong agreement between GAT, Tono-Pen, and ICare tonometers in measuring IOP in corneas with MyoRings, and this agreement appears to be independent of the CCT in corneas with MyoRings. The ICare was found to have clinically irrelevant underestimation of IOP.

## Figures and Tables

**Table 1:** Correlation of IOP measurements by GAT, Tono-Pen, and ICare tonometer (Pearson correlation coefficient).

	GAT-Tono-Pen	GAT-ICare	Tono-Pen-ICare
*R*	0.889	0.710	0.712
*p* value	0.000	0.001	0.001

**Table 2:** Correlation of CCT with mean IOP difference between GAT, Tono-Pen, and ICare tonometer (Pearson correlation coefficient).

	GAT-Tono-Pen	GAT-ICare	Tono-Pen-ICare
*r*	0.056	0.247	0.232
*p* value	0.830	0.339	0.360

## Data Availability

All the spreadsheets, Pentacams, and data files of all patients used to support the findings of this study are available from the corresponding author upon request.
